# East Asian climate response to COVID-19 lockdown measures in China

**DOI:** 10.1038/s41598-021-96007-1

**Published:** 2021-08-19

**Authors:** Sun-Seon Lee, Jung-Eun Chu, Axel Timmermann, Eui-Seok Chung, June-Yi Lee

**Affiliations:** 1 Center for Climate Physics, Institute for Basic Science, Busan, South Korea; 2 Pusan National University, Busan, South Korea; 3 Division of Atmospheric Sciences, Korea Polar Research Institute, Incheon, South Korea; 4 Research Center for Climate Sciences and Department of Climate System, Pusan National University, Busan, South Korea

**Keywords:** Climate sciences, Atmospheric science

## Abstract

The COVID-19 pandemic caused disruptions of public life and imposed lockdown measures in 2020 resulted in considerable reductions of anthropogenic aerosol emissions. It still remains unclear how the associated short-term changes in atmospheric chemistry influenced weather and climate on regional scales. To understand the underlying physical mechanisms, we conduct ensemble aerosol perturbation experiments with the Community Earth System Model, version 2. In the simulations reduced anthropogenic aerosol emissions in February generate anomalous surface warming and warm-moist air advection which promotes low-level cloud formation over China. Although the simulated response is weak, it is detectable in some areas, in qualitative agreement with the observations. The negative dynamical cloud feedback offsets the effect from reduced cloud condensation nuclei. Additional perturbation experiments with strongly amplified air pollution over China reveal a nonlinear sensitivity of regional atmospheric conditions to chemical/radiative perturbations. COVID-19-related changes in anthropogenic aerosol emissions provide an excellent testbed to elucidate the interaction between air pollution and climate.

## Introduction

**Figure 1:**
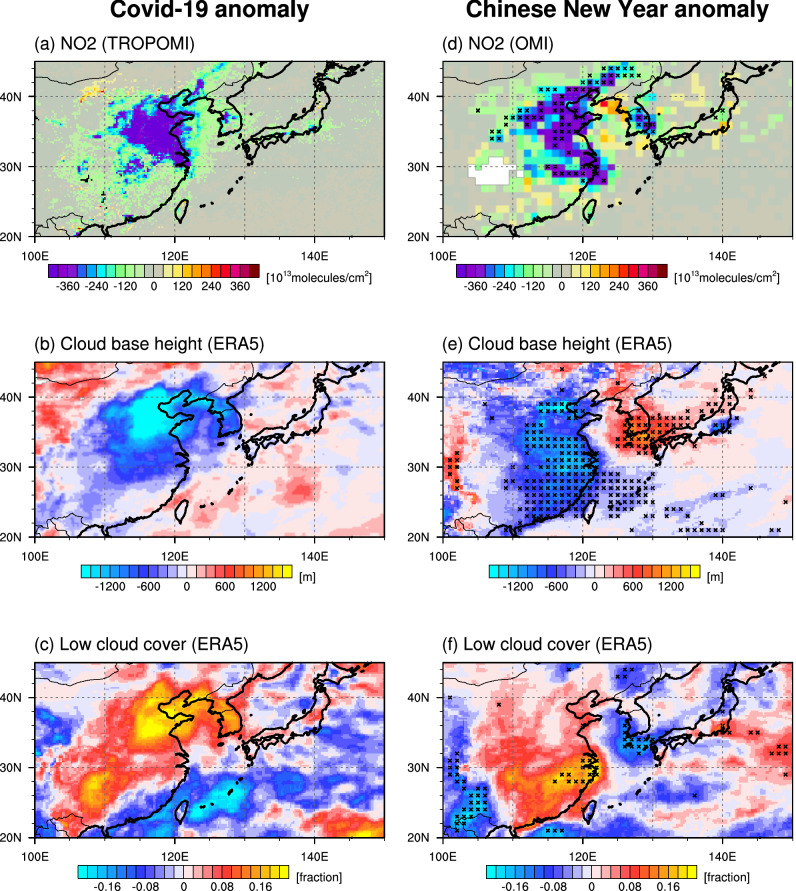
Anomalies of NO_2,_ cloud base height, and low cloud cover. (**a**) Difference between monthly NO_2_ concentration in February 2020 and the February composite over years 2018–2019 from TROPOMI^43^. (**b**) Difference between monthly cloud base height in February 2020 and the February composite over years 2016–2019 from ERA5^44^. (**c**) Same as in (**b**), but for low cloud cover. (**d**) Composite mean difference between OMI NO_2_ concentration^45^ for the week including Chinese New Year’s day and the previous week over years 2011–2020. (**e**) Same as in (**d**), but for cloud base height from ERA5. (**f**) Same as in (**d**), but for low cloud cover from ERA5. In (**d**–**f**), black crosses represent grid points where the composite mean difference is significant above the 90% confidence level. The figure was generated using NCAR Command Language Version 6.5.0 (http://dx.doi.org/10.5065/D6WD3XH5).

To contain the spread of the novel coronavirus SARS-CoV-2 (COVID-19), massive public health interventions were implemented in China from January 2020^1^. As a result, large parts of the manufacturing sector in China halted temporarily, causing a widespread reduction of anthropogenic aerosol emissions after the Chinese New Year holidays (also known as the Spring Festival). This is illustrated by the rapid drop in atmospheric NO_2_ concentrations in February 2020 relative to previous years, which attained values of 40–70% (Fig. 1a)^2–4^. The overall slowdown of emissions in the transportation, power, and industry sectors also led to a decline of total CO concentrations^3^ and an decrease in Aerosol Optical Depth (AOD) at 550 nm^5^ (Fig. S1). Consequently, during the COVID-19-related lockdown, air quality across China was improved on average^6^. However, this change appeared to be spatially inhomogeneous with some areas reporting anomalously high air pollution^7^.

Aerosols are known to alter the energy budget through aerosol-radiation and aerosol-cloud interactions^8–10^. An abrupt reduction or increase of anthropogenic aerosol emissions could therefore affect weather and climate. To quantify potential effects of the COVID-19 lockdown measures on current and future climate, coordinated earth system model experiments have been conducted^11–13^. The main conclusion from these perturbation simulations is that the aerosol reduction effects on climate are insignificant or very small on global scale. This might be because the emission reductions are not enough in magnitude and time to have a strong effect on a global climate^13^. On the other hand, widespread warming over parts of eastern China, South Asia, Europe, and the eastern United States occurs in the emission reduction simulations due to the fast climate response to aerosol reductions^14^. The ongoing and controversial discussion in recent studies highlights how difficult it is to deduce the magnitude of aerosol effects on climate^15–18^ from model simulations as well as from short observational datasets, in particular, in the presence of strong synoptic weather noise^19^. Whether the overall reduction of certain types of anthropogenic aerosols influenced regional weather and climate conditions still remains unclear, thus analyses on regional scales are required to understand the impact of COVID-19-related emission^13^.

Furthermore, resolving the aerosol effects on weather and climate is necessary for understanding how future implementation of air pollution mitigation strategies nationally and/or internationally will impact regional and large-scale climate conditions^20,21^. The unusual aerosol conditions in early 2020 in China and other parts of the world subsequently, could serve as a testbed to benchmark the performance of state-of-the-art earth system models and their representations of aerosol-radiation and aerosol-cloud interactions. It is therefore paramount to determine whether temporary lockdown measures in China from late winter to spring of 2020 impacted air quality, weather, and climate patterns across East Asia. Hence, we conduct a suite of idealized aerosol perturbation ensemble sensitivity experiments using the Community Earth System Model (CESM) and compare the main results to observations to elucidate whether the COVID-19 lockdown measures impacted East Asian climate.

## Methods

### Experimental setup

To determine the influence of a short-term reduction in anthropogenic aerosol emissions on regional climate over East Asia we employ the CESM version 2.1.2^22^ with ~ 1° × 1° atmospheric and oceanic horizonal resolutions. There are 32 atmospheric vertical levels and 60 oceanic levels. We conduct a control experiment (CTL) and two aerosol perturbation experiments. The aerosol perturbation experiments include a weak anthropogenic aerosol emission experiment (W_AER) and a strong anthropogenic aerosol emission experiment (S_AER).

To prepare the initial conditions for these sensitivity experiments, we perform a 60-year-long present-day spin-up simulation with annually repeating anthropogenic aerosol emissions^23^ and greenhouse gas concentrations^24^ corresponding to the 2010 CE observational estimates. We then identify the January sea surface temperature (SST) state that shows the closest resemblance (in a root mean squared error sense) between the spin-up simulation and the observed SST anomaly for January 2020 obtained from HadISST^25^ and ERSST^26^ datasets. The selected year of the spin-up run is then used as an initial condition for three experiments.

In the CTL simulation, we use the Coupled Model Intercomparison Project Phase 6 (CMIP6)‐based emission values (year 2010) of anthropogenic aerosols originating from energy, industry, residential, transportation, agriculture and waste sectors (i.e., Community Emissions Data System emissions)^23^. In the W_AER, we reduce local anthropogenic aerosol emissions over East Asia [20°–50° N, 100°–145° E] in February using the ratio between satellite-derived February NO_2_ concentrations in 2020 and 2018–2019 (Fig. S2a). We change both aerosol mass and numbers for all anthropogenic aerosol types, and this modified forcing is interpreted here as proxy for the COVID-19-related reduction in anthropogenic aerosol emissions. For March and April, we apply a similar procedure to the CESM anthropogenic aerosol emissions worldwide. In the S_AER, instead of reducing the aerosol emissions by the observed NO_2_ ratio, we multiply the NO_2_ ratio pattern by a factor of five (Fig. S2b). This idealized experiment will shed light on the potential nonlinearity of the climate response to air pollution.

Starting from initial conditions on January 1st we conduct a 20-member ensemble of 1-year-long integrations for each experiment, i.e., CTL, W_AER, and S_AER. Slightly different (~ 10^–14^ K) atmospheric temperature initial conditions (micro-perturbations) are applied for the ensemble runs. This approach overcomes potential difficulties in identifying aerosol-cloud interactions statistically from limited observational datasets which exhibit large unforced variability in cloud properties^19^; but it relies on the specific parameterizations of aerosol-cloud microphysics processes in the CESM.

### Conditional composite

Since synoptic weather conditions may influence the regional climate response to aerosol forcings, we pay extra attention to the realism of initial and boundary conditions by applying conditional composites. To improve the detectability of the forced atmospheric signals in the sensitivity experiments relative to the internal noise, we select a posteriori similar snow depth conditions in the ensemble sensitivity experiments and the CTL and compare the respective differences in meteorological variables between the perturbation runs and CTL. The conditional composite is applied only to snow depth in February. The underlying assumption is that the aerosol forcing in February itself does not affect the snow cover in the same month. Without this pre-selection, randomly occurring differences in the snow distribution would generate strong shortwave flux anomalies and in turn local atmospheric circulation differences among the ensemble members and the experiments^27^. We compare snow depth anomalies over Asia [Eq.–50° N, 60°–140° E] in each ensemble member of the perturbation experiment with the CTL simulation and choose only ensemble members that are similar to those in the CTL simulation based on pattern correlation coefficients. We only consider these selected ensembles in the composite analysis. In addition, although we apply fractional aerosol forcings from February to April (Fig. S2), we focus our analysis on February and the East Asian region.

## Results

### Observed cloud response to aerosol reduction: COVID-19 lockdown vs. Chinese New Year holidays

Regular or occasional reductions of the human and industrial activities can change the concentration of anthropogenic air pollutants. For example, similar to the COVID-19 lockdown, the extra measures taken during the Beijing Olympic Games in summer 2008 decreased PM2.5 (aerosol particulate matter with diameters smaller than 2.5 µm) by 48–56% over northeastern China^28^. Likewise, the station-based observations show a significant reduction of air pollution concentrations around the Chinese New Year holidays^29^. Thus, to examine the potential impact of aerosol emission reductions in China on East Asian climate and particularly on clouds, we compare the changes of aerosol concentrations and low-level clouds in February 2020 (the primary COVID-19 lockdown period in China) with the weekly composite difference between conditions after and before the Chinese New Year holiday. The composite is based on the periods from 2011 to 2020. Although China implemented lockdown measures in Wuhan already on January 23, 2020^30^, these were quite localized initially. Thus, the inclusion of the year 2020 in the composite of Chinese New Year holidays does not affect our conclusions.

Both for the February 2020 case and the Chinese New Year holiday, we find a substantial reduction of anthropogenic aerosols (as exemplified by the reduced column-integrated NO_2_ concentrations) over the eastern China (Fig. 1a,d)—one of the most populated and rapidly developing regions in the world. In addition, we find during the Chinese New Year a statistically significant (> 90%, see Supporting Information, Text S1) drop of the cloud base height and a large-scale increase in low-level cloud cover over the most industrialized areas in eastern China (Fig. 1e,f). In the composite difference between the COVID-19 lockdown phase in February 2020 and previous years (2016–2019), we find a decrease in cloud base height and an increase in low-level cloud cover over China, although the strongest anomalies occured in northeastern China (Fig. 1b,c). Differences in the cloud patterns between the COVID-19 case and the Chinese New Year holidays can be partly explained by the fact that the Chinese New Year holiday composite is based on a 10-year average, which reduces noise due to synoptic transients more effectively than for the single-realization of COVID-19 lockdown composite.

Whether the climatic anomalies that occurred in February 2020 over parts of East Asia, which included, among others, a decrease of the cloud base height by up to 2 km and an increase of low-level cloud cover by 0.1–0.2 over industrialized northeastern China and parts of the Korean Peninsula can be directly linked to the imposed reduction of air pollution remains an open question. In fact, a potential increase in cloud cover in response to a reduction of anthropogenic aerosols would seem counter-intuitive to our understanding of the hygroscopic properties of aerosols serving as cloud condensation nuclei (CCN).

### Reduced anthropogenic aerosols and cloud changes

**Figure 2:**
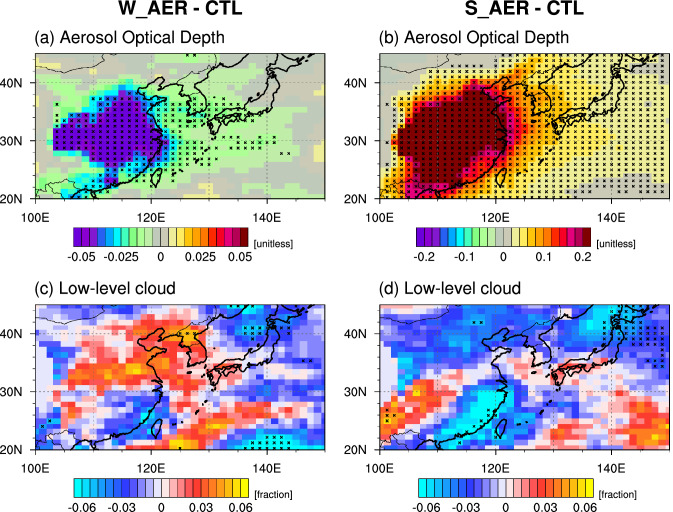
Difference fields in February between W_AER and CTL (left panels) and between S_AER and CTL (right panels). (**a**,**b**) Aerosol optical depth and (**c**,**d**) low-level cloud. Black crosses represent grid points where the composite mean difference is significant above the 90% confidence level. The figure was generated using NCAR Command Language Version 6.5.0 (http://dx.doi.org/10.5065/D6WD3XH5).

To better understand the robustness of the observational results (Fig. 1) and identify the underlying mechanism of increased low-level cloud cover during times of reduced anthropogenic aerosol concentration, we compare simulated low-level clouds in the W_AER with the CTL. As expected from the imposed 40–70% reduction of aerosol loadings in the W_AER we find a substantial decrease in AOD over China (Fig. 2a), which bears qualitative resemblance to the observations (Fig. S1b). As a result, the concentration of CCN decreases substantially by ~ 30%, particularly in the lower troposphere (Fig. S3a). However, similar to the observations, we find a weak increase in low-level cloud cover in northeastern China, which extends across the Korean Peninsula (Fig. 2c). This seemingly counter-intuitive result is also reflected in the vertical profile of cloudiness in W_AER (Fig. S3c), even though the response is quite weak and not statistically significant. Furthermore, consistent with the observations (Fig. 1c), the model also simulates a reduction of low-level clouds over southern China. Again, the amplitude is reduced relative to the observations.

To explore the robustness of the cloud response and potential nonlinearity of climate responses to aerosol forcings, we further investigate the AOD and cloud response in the S_AER, which applies unrealistically high anthropogenic aerosol emissions. As expected, AOD increases substantially over China (Fig. 2b). As the aerosol emissions increase, CCN increases linearly by threefold in the lower troposphere (Fig. S3b) compared to CTL case. Despite this massive forcing, we only find a very weak and statistically insignificant cloud anomaly over China (Fig. S3d), but a more robust downstream response over northern Japan (Fig. 2d).

### Circulation changes driven by aerosol direct effect

To better explain why the drastic changes in CCN in W_AER and S_AER do not translate into larger changes in cloud cover, we hypothesize here that the radiative effect induced by changes in aerosol concentrations leads to anomalous surface warming or cooling, respectively, causing low and high sea level pressure anomalies. This in turn leads to anomalous moisture advection towards China and subsequent changes in clouds^31^, potentially offsetting the direct CCN effect.

**Figure 3:**
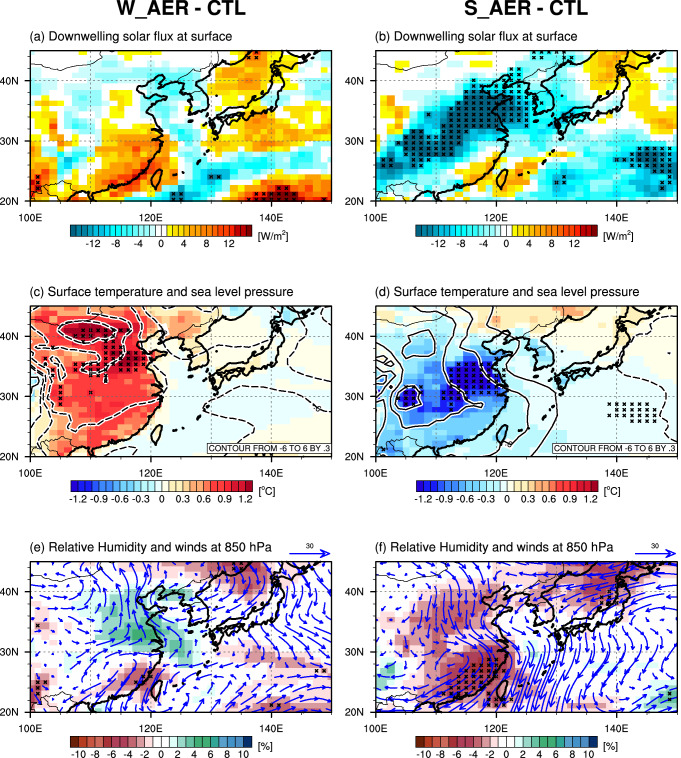
Difference fields in February between W_AER and CTL (left panels) and between S_AER and CTL (right panels). (**a**,**b**) Downwelling solar flux at surface, (**c**,**d**) surface temperature (shading) and sea level pressure (contour), and (**e**,**f**) relative humidity (shading) and wind (vector) at 850 hPa. Black crosses represent grid points where the composite mean difference is significant above the 90% confidence level. The figure was generated using NCAR Command Language Version 6.5.0 (http://dx.doi.org/10.5065/D6WD3XH5).

**Figure 4:**
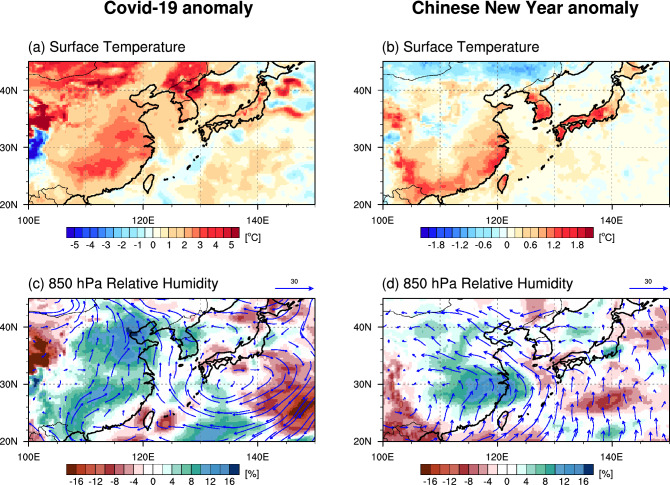
Difference fields between February 2020 and the February composite over years 2016–2019 (left panels) and composite mean between the week including Chinese New Year’s day and the previous week over years 2011–2020 (right panels). (**a**,**b**) Surface temperature and (**c**,**d**) relative humidity (shading) and wind (vector) at 850 hPa. The figure was generated using NCAR Command Language Version 6.5.0 (http://dx.doi.org/10.5065/D6WD3XH5).

To test this hypothesis, we examine the possible contribution of aerosol-induced changes in circulation and moisture transport to cloud formations. In the W_AER, the reduced AOD leads to an increase in downwelling shortwave radiation (Fig. 3a), resulting in anomalous surface warming over China (Fig. 3c)^32^. This process can destabilize the atmosphere and enhance vertical mixing^33^. Even though we observe the AOD reduction in most parts of China, the increased downwelling shortwave radiation is most pronounced in southeastern China. This mismatch can be explained by increased low-level cloud cover in northeastern China as shown in Fig. 2c and the corresponding cloud radiation properties. Increased shortwave radiation leads to surface warming, lower sea level pressure and southeasterly winds (Fig. 3c,e). This anomalous atmospheric circulation transports more moisture from the adjacent ocean to central and northeastern China. Consequently, low-level relative humidity increases in northeastern China (Fig. 3e), which further promotes the formation of low-level clouds, thereby offsetting the effect from reduced CCN concentrations. Furthermore, the anomalous circulation also leads to warm air advection, which explains why the maximum surface warming does not coincide spatially with the strongest radiative heating. Interestingly, we note that observed composite differences in February 2020 and for the Chinese New Year holidays also exhibit a similar relationship between surface temperature, circulation and clouds. In both cases, the aerosol direct effect by reduced aerosol concentrations leads to anomalous surface warming (Fig. 4a,b), which is accompanied by southerly winds, and increases in relative humidity (Fig. 4c,d). This analysis highlights that the aerosol direct effects may trigger atmospheric circulation changes which compensate the initial CCN effect in clouds. The net cloud response will eventually be determined by a delicate balance of two compensating effects. The efficiency of this compensation mechanism could be further affected by the prevailing seasonal circulation and atmospheric stratification.

Despite using an ensemble simulation approach and applying conditional composite, the W_AER results are only weakly statistically significant. This may imply that the COVID-19-related aerosol forcing is relatively weak to generate a significant climate response^13^ even on regional scales. Owing to the strongly imposed perturbation, the S_AER simulations show a more robust response: enhanced aerosol loadings reduce downwelling shortwave radiation, leading to surface cooling, anomalous high-pressure and northerly winds (Fig. 3b,d,f). This circulation pattern advects dry and cold air over East Asia, which in turn reduces clouds formation (Fig. 2d), in accordance with a previous study^34^. Also, here we observe the strong compensation between CCN effects and circulation on clouds.

In the W_AER experiment, we find a 30% reduction of CCN concentrations over China, whereas for the S_AER simulation, there is an intensification of 300% (Fig. S3a,b). From these initial forcings, we would expect a widely different magnitude in the responses. However, the regional signal does not scale linearly with the forcing. We find a warming of about 0.5–1.0 °C over the central part of eastern China in W_AER and cooling of only − 1.0 to −1.5 °C in S_AER (Fig. 3c,d). This saturation of the response might be another supporting evidence for a strong CCN/circulation compensation mechanism for cloud formation at work. To better understand these processes and the fact that the observed surface temperature response was even stronger (~ 4 °C) (Fig. 4) than the simulated one, one needs to further analyze the role of lateral heat advection versus radiative anomalies and their relationship to the underlying cloud feedbacks.

## Discussions and conclusions

Our study presents an observational and model-based analysis of the East Asian atmospheric conditions that occurred in conjunction with the COVID-19-related lockdown measures over China in February of 2020. By running a 20-member ensemble of anthropogenic aerosol emission sensitivity experiments, in combination with a conditional composite, we were able to distinguish the forced response from the internal atmospheric noise.

In the COVID-19 lockdown sensitivity experiment, W_AER, which mimics the observed reduction in anthropogenic aerosol emissions over China in February, we find a weak increase in low-level cloud cover and this is qualitatively consistent with the observations in February 2020 and Chinese New Year holidays (Fig. 1). Our study suggests that the aerosol direct radiative effect (reduction of AOD and increased downwelling shortwave radiation) (Figs. 2a, 3a) plays a key role in the initial surface warming, which in turn drives a low-pressure anomaly (Fig. 3c). The corresponding anomalous southerly circulation advects warm and moist marine air across parts of eastern China (Fig. 3e), leading to an enhancement of surface warming in some areas and an increase of relative humidity and low-level cloud cover (Fig. 2c). A similar feature, albeit much stronger and extending further south, can be found in the observations (Fig. 4).

Differences between the observations and the weaker model-simulated response may stem from a variety of factors, including initial conditions, influence of weather noise, limited statistics in the observational cases, idealized aerosol forcing in perturbation experiments, which affect all anthropogenic emissions equally, and uncertainties in the parameterization of cloud-aerosol interactions in CESM. Our study highlights the importance of a negative cloud feedback which invokes the dynamical atmospheric response to initial direct aerosol forcing (Fig. S4). In essence, reduced CCN concentrations can eventually cause an increase in cloud cover due to anomalous moist air advection. The stability of this feedback with respect to changes in seasonality and the amount of aerosol forcing needs to be carefully considered in future studies. In addition, in the present study, we utilize cloud data from ECMWF's Fifth generation reanalysis (ERA5). Even though the ERA5 represents the spatiotemporal distribution of cloud cover from satellite-based observations reasonably well^35,36^, there remain important uncertainties which need to be further assessed and studied.

Additional experiments (S_AER), which apply idealized and unrealistically strong air pollution over China, show a climate response that is comparable in magnitude (opposite in sign) to that of the much weaker forcing simulation (W_AER). This result further underscores the complexity and potential nonlinearity of the negative dynamical cloud feedback, which operates in late winter in East Asia. According to the simulations presented here, air pollution in boreal winter has the tendency to strengthen the East Asian winter monsoon, in agreement with previous studies^37^. However, in summer the aerosol direct radiative effect would cool the surface, weakening the land-sea thermal contrast and resulting pressure gradients. This would translate into a weakening of the East Asian summer monsoon, as proposed recently^38–41^.

In 2020 many other countries imposed similar lockdown measures to battle the spread of the COVID-19 disease. Moreover, global transportation decreased, which together led to a temporary improvement of global air quality in 2020^42^. How regional-scale weather and climate conditions elsewhere responded to the changes in anthropogenic aerosol concentrations will be left for future work. What is clear is that the observational data collected during the periods of rapid and constrained anthropogenic radiative perturbations in 2020 will become an invaluable future resource to test and calibrate climate and atmospheric chemistry models, which in turn will help in determining the impacts of future long-term air pollution mitigation strategies on our climate system.

## Supplementary Information

Supplementary Information.

## Data Availability

The CESM codes are freely available online at www.cesm.ucar.edu:/models/cesm2/. We acknowledge the free use of tropospheric NO_2_ column data from the TROPOMI sensor from www.temis.nl. The satellite data used in this study consist of carbon monoxide concentration from AIRS/Aqua L3 (https://disc.gsfc.nasa.gov/datasets/AIRS3STM_006/summary?keywords=AIRS%20L3) and AOD at 550 nm using Suomi National Polar-orbiting Partnership (SNPP) Visible and Infrared Imaging Radiometer Suite (VIIRS) NASA standard Level-3 monthly deep blue aerosol data (https://ladsweb.modaps.eosdis.nasa.gov/missions-and-measurements/products/AERDB_M3_VIIRS_SNPP/) over years 2016–2020. Daily NO_2_ concentration is obtained from OMI (https://daac.gsfc.nasa.gov/datasets/OMNO2d_003/summary?keywords=%22Aura%20OMI%22) during the period 2011–2010. Meteorological data including surface temperature, sea level pressure, 850 hPa relative humidity, 850 hPa zonal and meridional winds, cloud base height, and low cloud cover are obtained from ERA5 (https://www.ecmwf.int/en/forecasts/datasets/reanalysis-datasets/era5).
