# Supplementary material for: East Asian climate response to COVID-19 lockdown measures in China
Source: Sci Rep. 2021 Aug 19;11:16852. doi: 10.1038/s41598-021-96007-1 (PMC8376968; doi:10.1038/s41598-021-96007-1)
Supplement: Supplementary file 1 — Supplementary Information. [file 41598_2021_96007_MOESM1_ESM.docx]

Supporting Information for

East Asian climate response to COVID-19 lockdown measures in China

**Sun-Seon Lee^1,2^, Jung-Eun Chu^1,2*^, Axel Timmermann^1,2^, Eui-Seok Chung^1,2,3^, and June-Yi Lee^1,4^**

^1^Center for Climate Physics, Institute for Basic Science, Busan, South Korea

^2^Pusan National University, Busan, South Korea

^3^Division of Atmospheric Sciences, Korea Polar Research Institute, Incheon, South Korea

^4^Research Center for Climate Sciences and Department of Climate System, Pusan National University

**Contents of this file**

Text S1

Figures S1 to S4

**Text S1.**

**Procedures of significant test for changes related to the Chinese New Year holidays**

(1) Randomly select two consecutive weeks (W1 and W2) between January 1^st^ and February 28^th^ for each year from 2011 to 2020

(2) Compute the weekly mean anomaly (because the difference between the two weeks might result partly from the climatology difference, the mean seasonal cycle over the period 2005-2020 is removed) for NO_2_ concentration, cloud base height and low cloud cover, respectively, for the two weeks (W1 and W2)

(3) Compute the difference (W2 minus W1)

(4) Compute the composite mean difference over the 10 years (2011 to 2020)

(5) Repeat the above steps (1) to (4) 9999 times

(6) At each grid point, the 10000 values are sorted out, respectively, for NO_2_ concentration, cloud base height and low cloud cover

(7) If the composite mean difference lies within the top 5% or bottom 5% of the range, then that difference is assumed to be statistically significant at the 90% confidence level


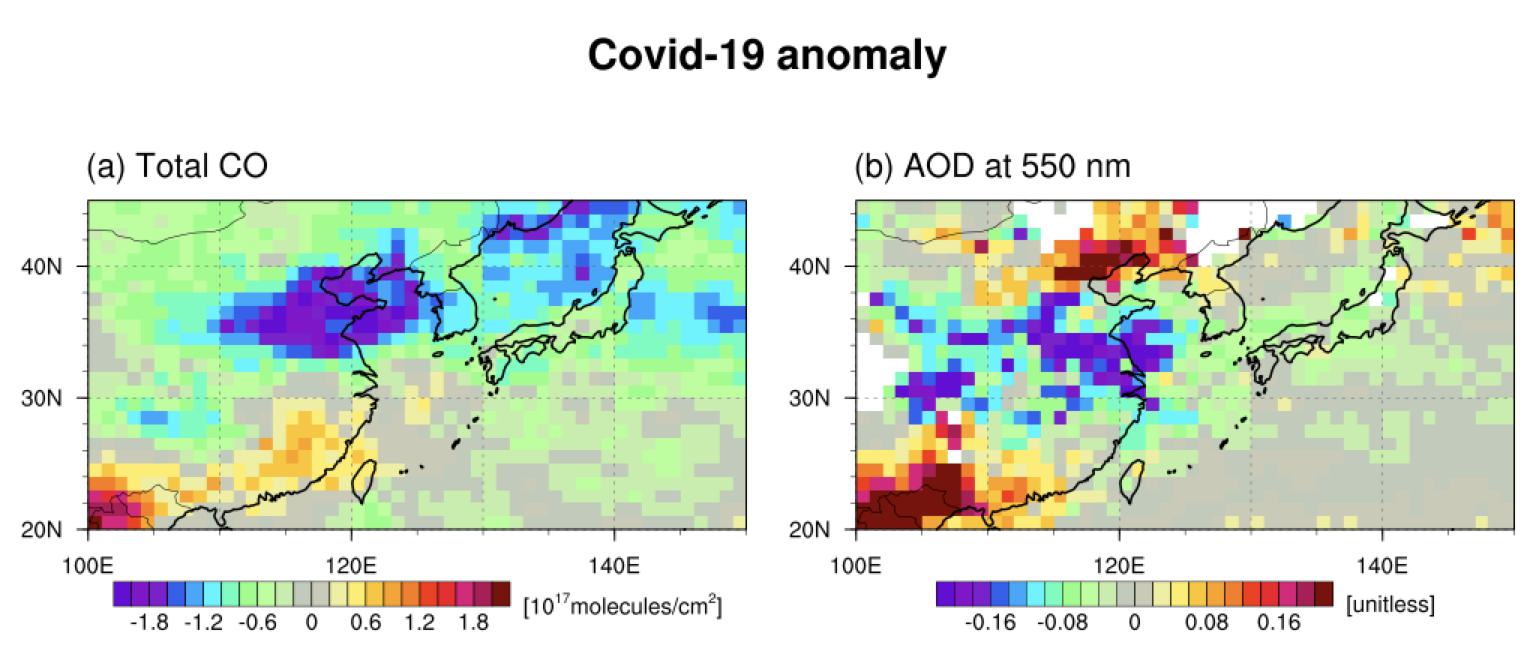


**Figure S1.** February 2020 anomaly relative to the corresponding month composite over years 2016-2019. (a) Total carbon monoxide concentration from AIRS/Aqua L3^1^. (b) Aerosol optical depth (AOD) at 550 nm using Suomi National Polar-orbiting Partnership (SNPP) Visible and Infrared Imaging Radiometer Suite (VIIRS) NASA standard Level-3 monthly deep blue aerosol data^2^. The figure was generated using NCAR Command Language Version 6.5.0 (http://dx.doi.org/10.5065/D6WD3XH5).


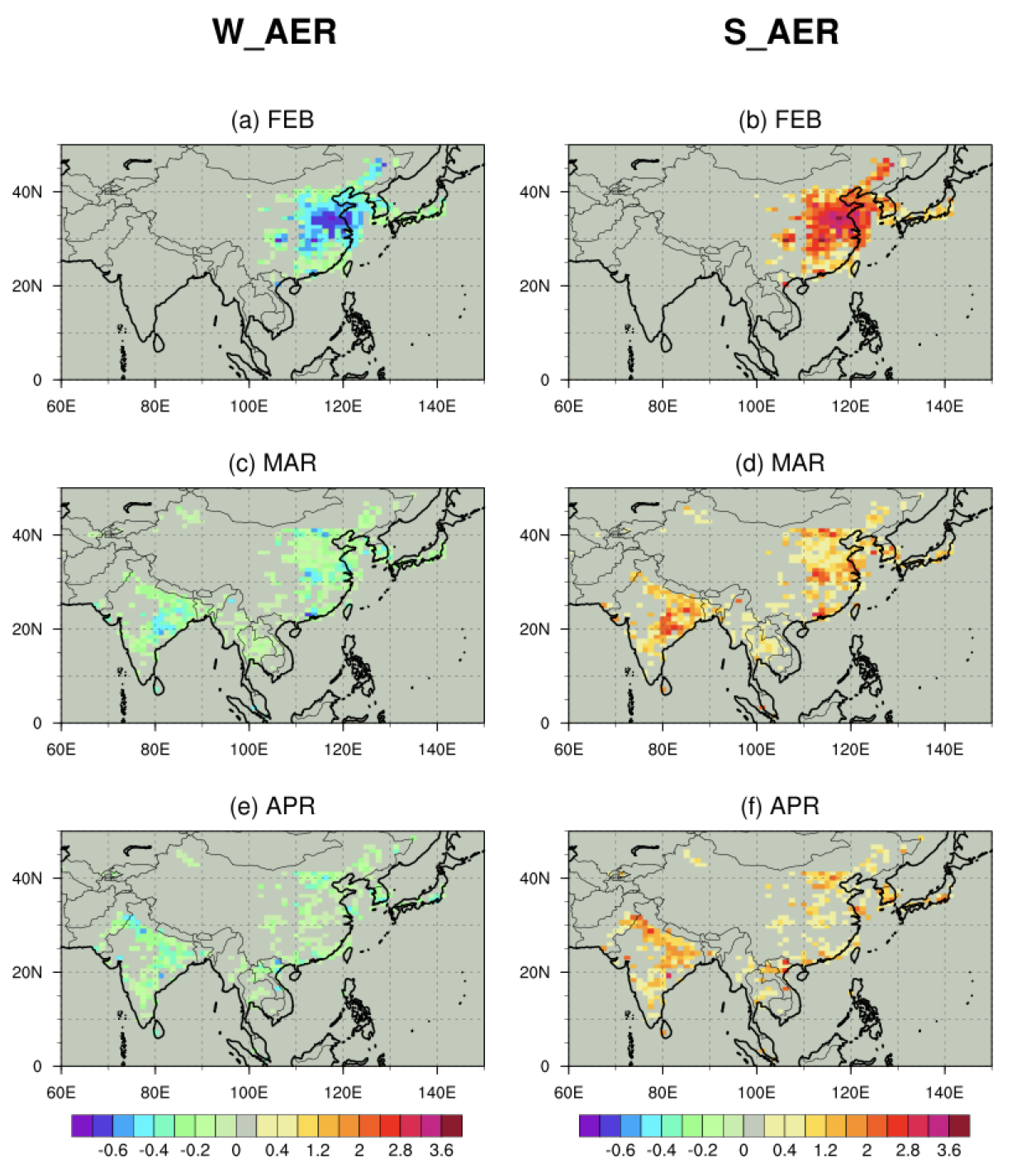


**Figure S2.** Fractional change (*frac*) of anthropogenic aerosol emissions used in W_AER (left panels) and S_AER (right panels). (a)(b) February, (c)(d) March, and (e)(f) April. For W_AER experiment, anthropogenic aerosol emissions are calculated by multiplying ‘1+*frac*’ and original emission values that used in CTL. For S_AER experiment, anthropogenic aerosol emissions are calculated by multiplying ‘1+*frac*×5’ and original emission values that used in CTL. The figure was generated using NCAR Command Language Version 6.5.0 (http://dx.doi.org/10.5065/D6WD3XH5).


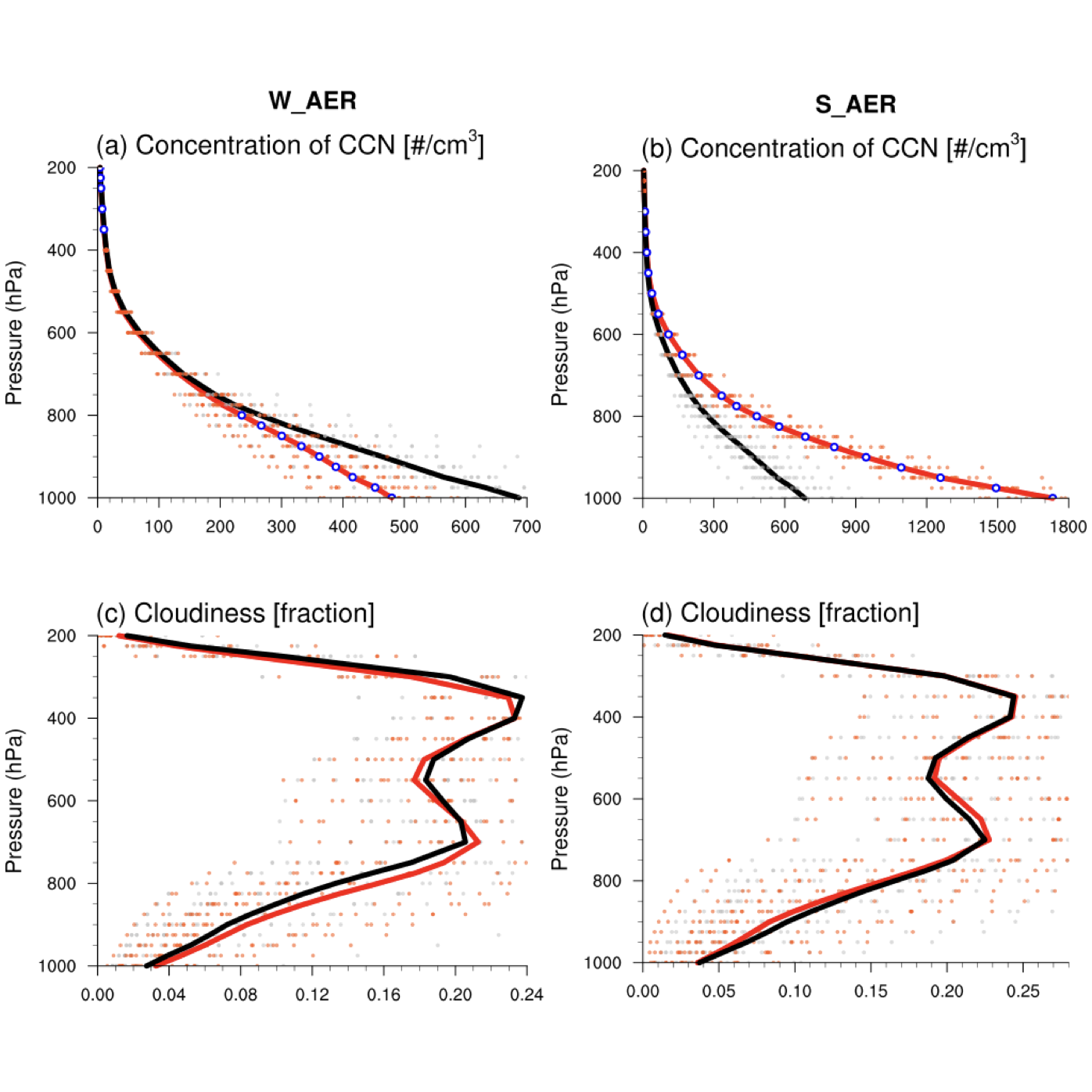


**Figure S3.** (a) Cloud condensation nuclei concentration as function of pressure in February averaged over northeast China (30°N-40°N, 110°E-120°E) for the CTL (gray dots: each ensemble, black line: ensemble mean) and W_AER (orange dots: each ensemble, red line: ensemble mean). (b) Same as (a), but for S_AER (orange dots: each ensemble, red line: ensemble mean). (c) Same as (a) but for cloudiness. (d) Same as (c) but for S_AER. Blue circles indicate statistically significant differences above the 90% confidence level. The figure was generated using NCAR Command Language Version 6.5.0 (http://dx.doi.org/10.5065/D6WD3XH5).


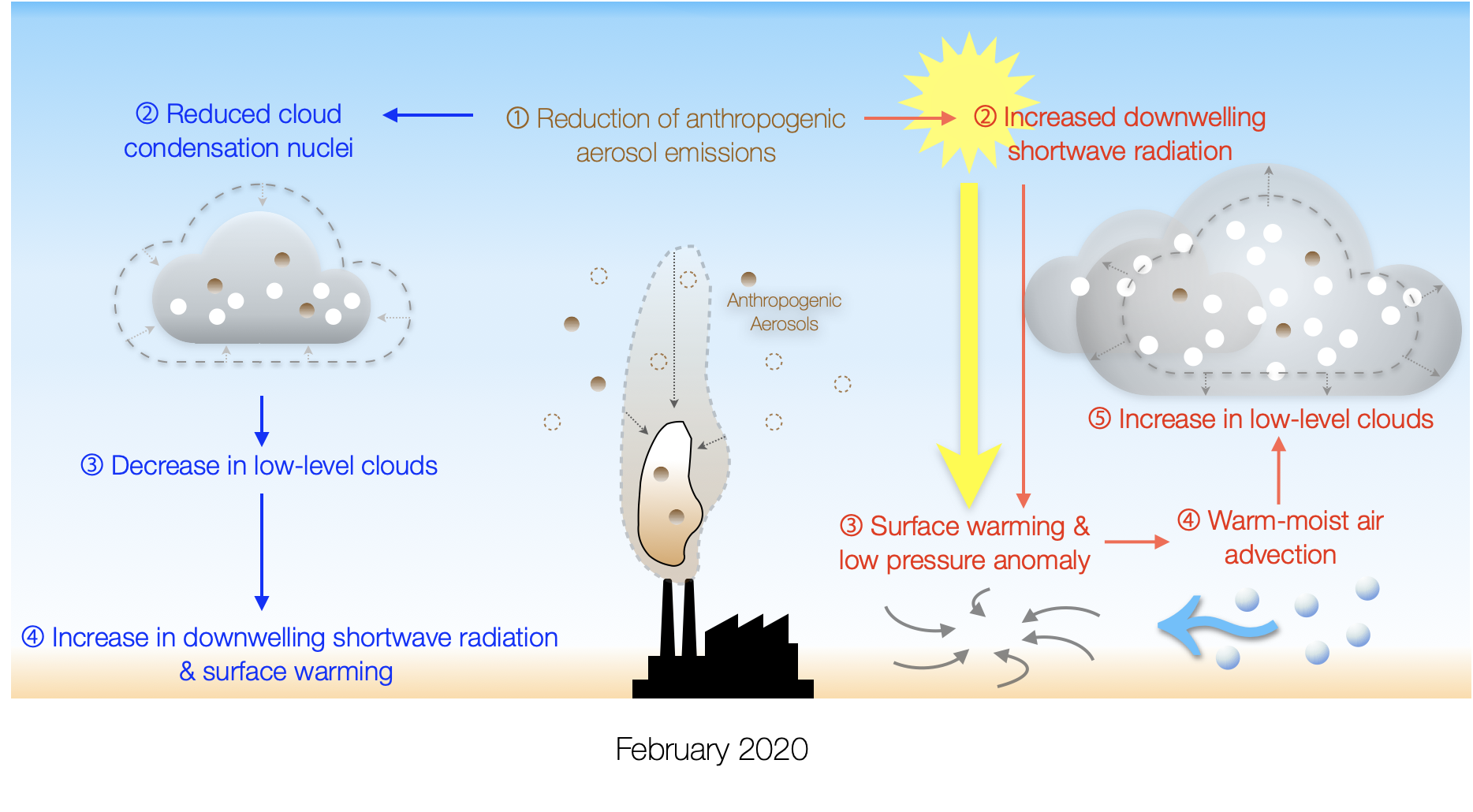


**Figure S4.** Schematic diagram illustrating cloud response to COVID-19-related reduction in anthropogenic aerosol emissions. Blue color shows the effect of reduced cloud condensation nuclei concentrations, resulting in decrease in low-level clouds. Red color shows the process of increase in low-level clouds through the aerosol direct effect and subsequent moist-warm air advection.

References

1 AIRS Science Team/Joao Teixeira, AIRS/Aqua L3 Monthly Standard Physical Retrieval (AIRS-only) 1 degree x 1 degree V006, Greenbelt, MD, USA, Goddard Earth Sciences Data and Information Services Center (GES DISC), 10.5067/Aqua/AIRS/DATA321 (2013).

2 Sayer, A. *et al.* Satellite Ocean Aerosol Retrieval (SOAR) Algorithm Extension to S‐NPP VIIRS as Part of the “Deep Blue” Aerosol Project. *Journal of Geophysical Research: Atmospheres* **123**, 380-400 (2018).
